# The Medico-Psychological Association and Their Nurses

**Published:** 1897-01-09

**Authors:** 


### EDITOR'S LETTER-BOX.

[Our Correspondents are reminded that prolixity is a great bar to publication, and that brevity of style and conciseness of statement greatly facilitate early insertion.]

### "THE MEDICO-PSYCHOLOGICAL ASSOCIATION AND THEIR NURSES."

Sir,—Your article of January 2nd is so inaccurate that it must not be allowed to pass unnoticed. The Medico-Psychological Association Handbook for Attendants on the Insane (nearly 9,000 copies of which have been sold), in its third edition, 1896, contains in an appendix the rules for the nursing certificate.

Your article merely gives (*d*) the scope of training—passing over entirely (*g*) the syllabus of subjects in which training is compulsory, including symptoms of disease and disorder (of the circulatory, respiratory, alimentary, excretory, and nervous systems); also nursing of the sick, management of the sick room, personal attention to the sick, observation of cases (charts, records, temperatures, &c.); appliances, &c (poultices, baths, sponging, &c.); emergencies, principles of management in fainting, hæmorrhage, fractures of bones, epileptic fits, apoplexy, &c. Surely, in justice to your readers, this should also have been quoted! In all asylums, where the proper training for this examination is undertaken, the attendants and nurses pass three months in the hospital or infirmary wards, and the clinical demonstrations of the lecturer are carried on in these wards, where also the practical part of the examination is held. This three months' hospital ward work will, no doubt, shortly become compulsory in all cases. In this asylum, from 1887 to 1891, we gave lectures, held classes and examinations, and issued our own certificates after examination at the end of the session. These we ceased to grant in 1891, on the establishment of the certificate of Medico-Psychological Association, for we recognised the greater value of the latter to the staff in seeking promotion elsewhere, and verily do I believe that in the future no asylum appointment of matron, chief nurse, head attendant, charge attendant, or charge nurse will be obtainable, unless the candidate is the possessor of this qualification. Therefore, those superintendents who refuse to encourage the members of their staff in this matter are doing them a great injustice. The Medico-Psychological Association can well afford to ignore the pen-ultimate and ultimate paragraphs of your article. The steadily increasing number of its members (there are now upwards of 500), and its ever-expanding work, educational and scientific, are a sufficient answer to those who would undervalue its sphere of usefulness.—I am, &c , ERNEST W. WHITE.

City of London Asylum, near Dartford, January 4th.

*** Our remarks were founded upon the report of the committee appointed to consider the training of nurses, which we received from an official of the Association. The syllabus of compulsory subjects does not appear in it, nor is it found in the first edition of the Handbook, which we presume was the text-book until the new edition in 1896 appeared. It is present in that edition, but so far as it refers to sick-room work does not differ materially from the syllabus for St. John's nursing certificates. We claim Dr. White's letter as corroboration of our statements. There is no compulsory period of training in the sick wards. How then can the practical experience be obtained? Dr. White's remarks about the excellent work done by the Association again bear out our contention that it should be brought within reach of the busy practitioner. We knew of Dr. White's excellent record in the City of London Asylum, and regret that he does not continue to put his own stamp on his finished work.—ED , *T. H.*

